# Clinical and neuropsychological characteristics of general paresis misdiagnosed as primary psychiatric disease

**DOI:** 10.1186/s12888-016-0925-3

**Published:** 2016-07-11

**Authors:** Wang Yanhua, Shi Haishan, Hou Le, Zhong Xiaomei, Chen Xinru, Li Ling, Wu Zhangying, Zheng Dong, Zhang Yuefen, Tan Yan, Luo Xinni, Liu Sha, Ning Yuping

**Affiliations:** Department of Neurology, Guangzhou Brain Hospital, Guangdong, China; Department of Psychiatry, Sun Yat-sen University, Guangzhou Brain Hospital, Guangdong, China; Department of Psychiatry, Guangzhou Medical University, Guangzhou Brain Hospital, Guangdong, China; Department of Radiology, Guangzhou Brain Hospital, Guangdong, China

**Keywords:** General paresis, Primary psychiatric disease, Misdiagnosis, TPHA, RPR

## Abstract

**Background:**

Neurosyphilis is caused by the invasion of Treponema pallidum into the central nervous system. General paresis (GP) is a type of neurosyphilis. The main manifestation of general paresis is dementia; however, this is different from the other types of dementia, which can be cured by adequate doses of penicillin in the early stage. Neurosyphilis is the “great imitator” because it can mimic many types of medical disorders. In addition, the manifestations of neurosyphilis are not typical. Psychiatric disorders as a cause of general paresis have become more common due to the use of antibiotics. Patients with a psychiatric manifestation are often misdiagnosed. The purpose of this study was to explore the differences in the clinical and neuropsychological characteristics of general paresis between patients misdiagnosed as having a primary psychiatric disease and patients diagnosed correctly upon seeing a doctor. The results may assist clinicians in the early identification of neurosyphilis with a mental disorder.

**Method:**

The demographic and clinical manifestations, laboratory tests, and neuroimaging and neuropsychological characteristics were analysed in 55 general paresis patients with psychiatric disorders, including 29 patients misdiagnosed as primary psychiatric disease and 26 patients diagnosed as having general paresis after being seen once by a doctor.

**Result:**

All of the patients had positive assay results for cerebral spinal fluid (CSF) Treponema pallidum hemagglutination (TPHA). Only 43.3 % of misdiagnosed patients and 30.8 % of general paresis patients had positive results for the CSF rapid plasma reagin (RPR) test; 96.4 % patients had abnormal neuroimaging. Mood disturbances were the most common psychiatric disorder in the general paresis patients, especially agitation, between the two groups (patients with general paresis who were misdiagnosed as having primary psychiatric disease and patients who had never been misdiagnosed) (*p* = 0.011).

**Conclusion:**

Our findings reinforce the importance of performing serologic testing for syphilis. This should be a part of the evaluation of patients with psychiatric disorders, especially patients with cognitive impairment. When the syphilis serology is positive, the patient should be examined thoroughly for neurosyphilis by lumbar puncture. Brain imaging could also aid the physician in discriminating these patients from those with a functional mental disorder.

## Background

Neurosyphilis (NS) is caused by the invasion of Treponema pallidum into the central nervous system, which can occur in any stage of syphilis, especially in the late stage [1]. In addition, up to 40 % of untreated patients progress to NS [2]. This includes asymptomatic NS, syphilitic meningitis, meningovascular syphilis, tabes dorsalis and general paresis (GP). GP is a type of dementia secondary to syphilis that can be cured by adequate doses of penicillin. Remission is determined by the severity and duration of the illness. Therefore, early diagnosis and early treatment are crucial. However, NS is known as the “great imitator” because it can mimic many other medical and neuropsychiatric disorders, including personality disorders, psychosis, and dementia [3]. Typical symptoms of NS, such as Argyll-Robertson pupil, VIII cranial nerve disorders and tabetic indicators, have diminished significantly in recent years due to the use of antibiotics [4]. In addition, NS is currently considered to be an almost forgotten disease, which leads to limited experience with its clinical manifestations for the majority of young physicians [5]. These reasons may cause difficulty in recognizing NS in clinical practice.

In GP, patients appear irritable, forgetful and experience personality changes, headaches, and changes in sleep habits in the early stage while emotional lability, impaired memory and judgment, disorientation, confusion, delusions, and occasionally seizures occur in the late stage as other types of dementia [6]. However, because antibiotics are widely used, psychiatric disorders have become the most common clinical manifestation for these patients [4]. However, patients with psychiatric symptoms in these populations are often misdiagnosed as having primary psychiatric disorders. The previous literature has not reported the characteristics of GP patients who were misdiagnosed as having primary psychiatric disorders. In this study, we examine the clinical and neuropsychological characteristics of general paresis that differ between patients misdiagnosed as having primary psychiatric disease and patients diagnosed correctly once they see a doctor. This information may assist clinicians in the early identification of NS with mental disorders.

## Method

The patients diagnosed with GP were recruited from March 2011 to May 2015. The diagnostic criteria for NS complied with the Centers for Disease Control (CDC) guidelines [7] and the European Guidelines [8]. The criteria included positive serologies and one or more of the following: positive cerebral spinal fluid (CSF) rapid plasma regain (RPR), positive CSF Treponema pallidum hemagglutination assay (TPHA) and increased CSF protein (protein >500 mg/L) or white blood cells (WBC) (WBC .10 × 10^6 cells/L), and an otherwise unexplained neurological manifestation consistent with NS. GP fulfilled the DSM-IV criteria of dementia. The history and treatment were used to determine whether the patient was misdiagnosed. The patients who were misdiagnosed as having primary psychiatric disease were our focus. These patients were diagnosed as having a primary psychiatric disorder, but their symptoms did not go into remission following use of psychotropic medication. In addition, their symptoms improved with adequate doses of penicillin and small quantities of psychotropic medication. This study was approved by the ethics committees of the Guangzhou Brain Hospital (Number 32, 2009). The participants’ relative or legal guardian signed the informed consent to participate in the study.

Each participant underwent clinical evaluation, including obtaining their history, physical and neurological examinations, neuroimaging and laboratory testing. Once the patients were identified as NS, we conducted the neuropsychological test and performed computed tomography (CT) or magnetic resonance imaging (MRI) scans in a week. The Mini Mental State Examination (MMSE) was used to evaluate cognitive function, and the cut-off scores were adjusted by education levels [9]. The Chinese version of neuropsychiatric inventory (NPI) was used to evaluate neuropsychiatric symptoms. The total score was calculated by the sum of the 12 items in the NPI as frequency*severity [10].

### Statistical analysis

The gender distribution between the groups was evaluated using a chi-squared test. The duration from the symptoms onset to correct diagnosis and MMSE were presented as the median and range (min, max). Differences between the groups were analysed using the nonparametric Mann–Whitney U test. The age, education and total score of the NPI were presented as the mean ± SD. Differences between the groups were analysed using the independent-samples *t* test. Spearman’s correlation was used to assess the correlation between cortical atrophy and hippocampus atrophy. All of the statistical analyses were performed using SPSS 17.0 (SPSS Inc., Chicago, IL, USA), and *P* values < 0.05 were considered statistically significant.

## Result

### Clinical characteristics

A total of 92 in-patients with NS were identified from the Guangzhou Brain Hospital. Seventy-six patients (82.6 %) had psychiatric symptoms. Twenty-nine patients (38.2 %) had been misdiagnosed as having primary psychiatric disease, including schizophrenia (15 patients), mania (6 patients), bipolar disorder (5 patients), acute stress disorder (1 patient), anxiety (1 patient), and psychomotor attack (1 patient). Twenty-six patients were definitively diagnosed once they saw a doctor. The remaining 21 patients were misdiagnosed with other types of diseases, such as Alzheimer’s disease, Lewy body dementia, ataxia, cerebral ischemia, encephalitis, epilepsy and substance abuse.

### Demographic and clinical characteristics

The demographic and clinical information for the participants with GP who were misdiagnosed as having a primary psychiatric disease and the patients diagnosed definitively once seen by a doctor is listed in Table 1. The percentage of the males between the two groups was similar. The age in patients misdiagnosed as having primary psychiatric disease was younger than the patients diagnosed definitively once seen by a doctor (*p* <0.001). The duration from symptom onset to confirmed diagnosis was longer in patients who were misdiagnosed, but the difference was not significant. Argyll-Robertson pupil is a characteristic of NS that was not found in the patients in our study. A positive sucking reflex was the only neurophysical sign in our patients, but there was not a significant difference between the two groups. All of our patients had positive serum results for TPHA and RPR. The abnormal rate of CSF in the misdiagnosed group was 62.1 % and was 38.5 % in the group diagnosed after seeing a doctor. These rates were significantly different (*p* = 0.08). All of the patients’ CSF WBC counts were normal. The CSF TPHA was positive in all of the patients, but only 43.3 % of misdiagnosed patients and 30.8 % of direct diagnosed patients were CSF RPR positive.

**Table 1:** The demographic and clinical information for the participants between GP misdiagnosed as primary psychiatric disease and the patients diagnosed definitely once seeing a doctor

Variable	GP misdiagnosed (n = 29)	GP not misdiagnosed (n = 26)	*p* value
Number of male	26 (89.7 %)	23 (88.5 %)	0.887
Age (years)	48.2 ± 8.4	55.0 ± 8.2	<0.001
Education (years)	8.9 ± 2.3	8.7 ± 1.8	0.71
The duration (month)	16 (from 1 to 96)	12 (from 1 to 72)	0.054
Argyll-Robertson pupil	0 (0)	0 (0)	
Positive sucking reflex	12 (41.4 %)	12 (46.2 %)	0.721
CSF-protein (>500 mg)	18 (62.1 %)	10 (38.5 %)	0.08
CSF-WBC (>10*10^6)	0 (0)	0 (0)	
CSF-RPR (+)	14 (48.3 %)	8 (30.8 %)	0.186
CSF-TPHA (+)	29 (100 %)	26 (100 %)	

*Abbreviations*: *GP* misdiagnosed, general paresis misdiagnosed as primary psychiatric disease, *GP* not misdiagnosed, general paresis was diagnosed once seeing a doctor, The duration, the symptoms onset to correct diagnosis, *CSF* cerebral spinal fluid, *WBC* white blood cells, *RPR* rapid plasma regain, *TPHA* Treponema pallidum hemagglutination assay

### The neuroimaging characteristics

All of the patients underwent CT or MRI scans. In the 55 patients, 46 had MRI 3.0 T scans and 9 patients (4 in the misdiagnosed group and 5 in the directly diagnosed group) had CT scans. We found that 96.4 % of patients had abnormal neuroimaging. As shown in Table 2, cerebral atrophy was the most common abnormality seen in the patients in our study (86.2 % in the misdiagnosed group and 84.6 % in the control group). Subcortical white matter lesions were also common (79.3 % and 61.5 %) and were mainly focused in the frontal region, corona radiate, centrum ovals majus, or beside the lateral ventricle. There were no significant differences between the groups (*p* = 0.867, 0.147). Twenty-six patients had magnetic resonance spectroscopy (MRS) scans of the hippocampus (14 in the misdiagnosed group and 12 in the directly diagnosed group). We found that hippocampus atrophy and metabolic abnormality were common in the GP. The percentage of hippocampus atrophy in the two groups was 64.3 % and 41.7 %, while the percentage of hippocampus MRS was 64.3 % and 33.3 %. No significant difference was found between the groups. The hippocampus atrophy showed a significant correlation with cerebral atrophy (*r* = 0.527, *p* = 0.006).

**Table 2:** The neuroimaging of the patients

Variable	GP misdiagnosed (n = 29)	GP not misdiagnosed (n = 26)	*p* value
cortical atrophy	25 (86.2 %)	22 (84.6 %)	0.867
subcortical white matter ischemic	23 (79.3 %)	16 (61.5 %)	0.147
Hippocampus atrophy	9 (64.3 %)	5 (41.7 %)	0.249
	(n = 14)	(n = 12)	
Hippocampus metabolism abnormity	9 (64.3 %)	4 (33.3 %)	0.116
	(n = 14)	(n = 12)	

*GP* misdiagnosed , general paresis misdiagnosed as primary psychiatric disease, *GP* not misdiagnosed, general paresis was diagnosed once see a doctor

### Neuropsychology characteristics

Thirty-seven patients underwent a neuropsychological assessment (19 misdiagnosed and 18 controls). The other 18 patients were discharged from the hospital before we conducted the assessment. The details are shown in Table 3. The MMSE score ranged from 0 to 23, with a median score of 11 in the misdiagnosed patients and a median score of 15 in the controls. There was no statistically significant difference between them. In the NPI assessment, we found that the total score was higher in the misdiagnosed group (36.6 ± 1.8) than in the directly diagnosed group (18.2 ± 3.0) (*p* = 0.021). We further investigated the symptoms in detail. We found that mood disturbances, such as agitation, depression, anxiety, apathy, and irritability, were common in all patients with GP, regardless of whether they were misdiagnosed. In the misdiagnosed group, the percentage of neuropsychiatric symptoms was higher than the control, especially agitation (*p* = 0.011).

**Table 3:** The neuropsychology of the patients

Variable	GP misdiagnosed (n = 19)	GP not misdiagnosed (n = 18)	*p* value
MMSE	11 (range 0 to 23)	15 (range 0 to 23)	0.417
Delusions	10 (52.6 %)	7 (38.9)	0.402
Hallucinations	8 (42.1 %)	5 (27.8 %)	0.362
Agitation	16 (84.2 %)	8 (44.4 %)	0.011
Depression	11 (57.9 %)	5 (27.8 %)	0.065
Anxiety	11 (57.9 %)	6 (33.3 %)	0.130
Euphoria	10 (52.6 %)	4 (22.2 %)	0.057
Apathy	13 (68.4 %)	9 (50 %)	0.254
Disinhibition	5 (26.3 %)	4 (22.2 %)	0.772
Irritability	12 (63.2 %)	10 (55.6 %)	0.638
Abnormal motor behavior	8 (42.1 %)	3 (16.7 %)	0.091
Nighttime behavior	10 (52.6 %)	5 (27.8 %)	0.124
Appetite and eating disorders	4 (21.1 %)	3 (16.7 %)	0.734
Total	36.6 ± 1.8	18.2 ± 3.0	0.021

*GP* misdiagnosed, general paresis misdiagnosed as primary psychiatric disease, *GP* not misdiagnosed, general paresis was diagnosed once see a doctor, *Total* the sum of the 12 items’ frequency*severity, *MMSE* Mini-Mental State Examination

## Discussion

All of the patients in our study suffered from GP. They had no personal or family history of psychiatric disorders. We found that up to 82.6 % of patients had psychiatric symptoms. With the widespread use of antibiotics, neuropsychiatric symptoms have become more common in NS, especially in general paresis, the late stage of NS [11]. In the 1980s, only 17 % of NS patients presented with psychiatric disorders [12]. Currently, mental disorders occur in approximately 85.7 % of patients [4]. Young psychiatric physicians may be inexperienced in recognizing NS. It was often misdiagnosed as a primary psychiatric disorder. In our study, 29 patients (38.2 %) with mental disorders had been misdiagnosed as having a primary psychiatric disease, including schizophrenia (15 patients), mania (6 patients), bipolar disorder (5 patients), acute stress disorder (1 patient), anxiety (1 patient), and psychomotor attack (1 patient). All of these were common psychiatric disorders in the psychiatry clinic. In the misdiagnosed group, neuropsychiatric disorder was more severe than in the control group. Mood disturbances, such as depression, anxiety, apathy, irritability and agitation, were common. In addition, the agitation is the most common mood disturbance. As we know, delusions and hallucinations are the critical diagnosis standard for schizophrenia; however, in patients with GP, these symptoms were relatively rare [8]. This is in agreement with our study. Roberts et al. also reported on 21 patients with NS presenting with psychiatric manifestations and suggested that agitation could serve as an indicator for the diagnosis of NS [13]. The average age was 48.2 years old in the patients who were misdiagnosed, which is older than the average age of the onset of primary psychiatric disorders. The mean age was older in patients who were not misdiagnosed (55 years old). Cognitive impairment was obvious in our study but was often overlooked by the physicians, as the relatives were more concerned about their psychiatric symptoms. We found that cognitive impairment was more severe in the misdiagnosed group than in the control group when conducting the MMSE, although it was not statistically significant. The psychiatric and neurological symptoms may present as a consequence of extensively damaged parenchyma in cortical regions of the brain [14]. The duration from onset to correct diagnosis was longer for the patients who were misdiagnosed. When the neurophysical examination was performed, the only positive sign in our patients was a positive sucking reflex. Argyll-Robertson pupil is a characteristic of NS in which the pupil is unresponsive to light but constricts with accommodation or convergence. This symptom was not found in the patients in our study. Research found that Argyll-Robertson pupil is not common in GP, but is more often observed in tabes dorsalis [15], and in our study, none of the patients had tabes dorsalis.

Generally, CSF abnormalities, including pleocytosis, elevated protein, RPR, and TPHA, are crucial for reliably assessing the clinical stage of NS [16]. We found that the treponemal-specific test for TPHA in CSF was positive in all of the patients in our study, while the nonspecific lipoidal tests for RPR had a positive rate of 48.3 % in the misdiagnosed group and 30.8 % in the patients diagnosed correctly once seen by a doctor. Over time, the sensitivity of the lipoidal test to detect untreated late syphilis diminishes [15]. Meanwhile, all of the patients in our study had GP, the late stage of NS. Although some patients had negative RPR results, we combined the clinical manifestations and diagnosed them as NS. As a result, we should perform the treponemal-specific test even if the screening test was negative. Although nonspecific lipoidal tests for RPR were an important index for the activity of NS, antisyphilitic treatment should be given even if the patient had negative RPR results.

Brain imaging is important for the differential diagnosis of GP, especially to distinguish GP from functional disease. Currently, most reports about the brain imaging of NS are nonspecific. Cerebral atrophy [17] and subcortical white matter lesions [8] are the most common abnormalities. In our study, only two patients had normal neuroimaging. One was misdiagnosed as having bipolar disorder after conducting CT scans. The other was screened because her husband was a GP in our department. These nonspecific characteristics cannot provide more information about whether NS should be considered. However, it can provide a sign that this is not a functional disorder. We also carried out hippocampal structure and MRS scans, which mimicked those of Alzheimer’s disease (AD) in the imaging, such as hippocampus atrophy [18, 19]. Our team study also found that patients with general paresis had Alzheimer-type biomarker Ab42 changes, while the asymptomatic neurosyphilis patients did not have this change [20]. The hippocampus MRS was conducted to analyse its metabolism. Our findings suggest that the neuronal loss was in the hippocampus. A recent report also found that patients with mixed HIV infection and NS had substantial metabolic changes in the bilateral hippocampus, despite the paucity of MRI abnormalities in the structural scans [21]. Upon further study, we found that the patients who had hippocampal atrophy also had cerebral atrophy. We believe that the hippocampal atrophy was the result of global brain atrophy, which can be distinguished from early AD where hippocampal atrophy was obvious in the early stage. As a consequence, some researchers consider the hippocampus as indicative of a poor prognosis of NS [21].

NS is the “great imitator” because it can mimic many types of medical disorders. Inexperienced physicians often have difficulty in recognizing NS due to its plentiful clinical manifestations. Most NS manifestations are potentially reversible [22], especially in its early stage. Considering the diversity of the clinical manifestations of NS, we should combine clinical symptoms, blood screening and CSF tests when carrying out a diagnosis of NS. As psychiatric symptoms become more common in the antibiotic era, we should pay attention to the patients whose psychiatric symptoms begin later in life and who have no personal or family history of psychiatric disorders. Although screening all psychiatric patients for syphilis has been thought to be too costly [23], we should screen the patients with a high risk. This would include individuals whose partner(s) have tested positive for syphilis, who abuse drugs and alcohol or who are engaging in commercial or coerced sex [24]. We should be more concerned about the cognitive functioning in patients with psychiatric disorders. Brain imaging, although not specific, also provides a method to discriminate NS from primary psychiatric disease.

### Limitation

Our study had a cross-sectional design. In additional, selection bias is of concern. The patients in our study all had GP, the late stage of NS. Therefore, whether neuropsychiatric disorders such as agitation are common in the early stage of NS is unknown. In addition, the data in some patients were incomplete.

## Conclusion

GP is a potentially treatable type of dementia, and early diagnosis and early treatments are crucial for prognosis. Neuropsychiatric symptoms are the most common symptoms, especially mood disturbances, such as agitation. When we come across a patient with agitation in middle and old age, we should pay attention to his cognitive function. Blood screening is essential for high-risk patients. Reliable assessments should combine clinical manifestations and blood and CSF laboratory tests. Brain imaging may also provide an indicator for the diagnosis of NS.
